# Topical Formulations Containing *Mentha piperita* for Wound Healing: A Scoping Review

**DOI:** 10.1002/cbdv.202503567

**Published:** 2026-01-31

**Authors:** Lorena Gonçalves Araújo, Beatriz Gomes Vila‐Nova, Afonso Gomes Abreu

**Affiliations:** ^1^ Programa de Pós‐Graduação em Ciências da Saúde, Universidade Federal do Maranhão São Luís Brazil; ^2^ Laboratório de Imunofisiologia Universidade Federal do Maranhão São Luís Brazil

**Keywords:** antimicrobial activity, biofilm inhibition, *Mentha piperita*, nanostructured systems

## Abstract

Skin wound infections represent a major clinical challenge, exacerbated by biofilm formation and the increasing prevalence of bacterial resistance. Essential oils, particularly those of *Mentha piperita*, are well recognized for their antimicrobial and wound‐healing properties. This scoping review mapped the available evidence on the use of topical formulations containing *M. piperita*‐derived products for wound healing, with a focus on formulation vehicles, concentrations, experimental models, and efficacy parameters. Following JBI and PRISMA‐ScR guidelines, a comprehensive search was conducted in PubMed, Scopus, Web of Science, and LILACS (2010–2025). Twenty studies met the inclusion criteria, the majority of which were preclinical, comprising in vitro (35%), in vivo (30%), and combined in vitro/in vivo (30%) models. Only one ex vivo study was identified, and no clinical trials were found. The formulations investigated ranged from creams and gels to nanoemulsions, lipid carriers, films, and nanofibers, with concentrations varying from 0.5% to 20%. *Staphylococcus aureus* was the most frequently tested strain and showed greater susceptibility to peppermint essential oil (PEO). Incorporation into nanostructured systems was associated with improved stability, enhanced skin permeation, and superior antimicrobial performance, particularly when combined with nanoparticles, metals, other bioactive compounds, or, in specific cases, clinical antibiotics such as levofloxacin. Across both infected wound models and noninfected wound‐healing models, *M. piperita*‐containing formulations were associated with favorable outcomes, although the underlying mechanisms differed depending on the presence or absence of microbial challenge. Despite these promising findings, significant gaps remain regarding ex vivo validation, safety assessment, and clinical translation. Overall, PEO demonstrates considerable potential as an antimicrobial and wound‐healing agent; however, more standardized and clinically oriented studies are required.

## Graphical abstract

This scoping review synthesizes evidence from preclinical studies on topical formulations containing *Mentha piperita* for the treatment of infected wounds. The formulations, including creams, hydrogels, nanoemulsions, and lipid carriers, demonstrated antimicrobial and healing potential, reducing bacterial growth, biofilm formation, and inflammation, in addition to promoting re‐epithelialization. The evidence highlights the need for standardization and advanced biological validation

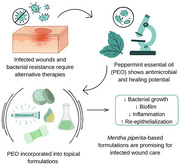

**Abbreviations**
AgNPs: silver nanoparticles
Au: gold
CMC: carboxymethylcellulose
*CTNNB1*
: catenin beta‐1
EGF: epidermal growth factor
FGF‐2: fibroblast growth factor‐2
GSH: glutathione
IL‐1β: interleukin‐1 beta
*M. piperita*
: *Mentha piperita*
MBC: minimum bactericidal concentration
MDA: malondialdehyde
MIC: minimum inhibitory concentration
MME: methanolic extract of *M. piperita*
MMP9: matrix metalloproteinase‐9
NC: nanocomposite
NLC: nanostructured lipid carriers
PCL: poly(ε‐caprolactone)
PEO: peppermint essential oil
PDI: polydispersity index
PVA: polyvinyl alcohol
qRT‐PCR: quantitative reverse transcription polymerase chain reaction
RT‐PCR: reverse transcription polymerase chain reaction
TGF‐β: transforming growth factor‐beta
TNF‐α: tumor necrosis factor alpha
WNT4: wingless‐type MMTV integration site family member 4
ZOI: zone of inhibition
in vitro: assay under controlled laboratory conditions
ex vivo: assay using excised tissue samples
in vivo: assay performed in living organisms.

## 1 Introduction

The skin, the largest organ of the human body, plays essential roles in thermoregulation and in maintaining fluid and electrolyte balance [1]. The integrity of this cutaneous barrier can be disrupted by trauma, burns, surgical procedures, or chronic diseases, resulting in wound formation [2]. During the healing process, injured skin is naturally colonized by microorganisms originating from the environment or the resident microbiota. However, clinical infection develops when host defense mechanisms fail to control bacterial proliferation, enabling tissue invasion, intensifying the inflammatory response, and delaying tissue repair [3].

Synergistic interactions among different bacterial species may increase pathogenicity under certain conditions, thereby exacerbating tissue damage and impairing host recovery. In such cases, colonization by pathogenic bacteria is directly associated with wound chronicity [4]. Chronic wounds may progress from contamination to colonization and, in some cases, from localized infection to systemic infection, sepsis, and multiple organ dysfunction syndrome [3].

Chronic wounds represent a significant public health burden, affecting an estimated 1%–2% of the global population and contributing substantially to morbidity and mortality [5]. The presence of pathogenic microorganisms, particularly in association with biofilm formation, promotes persistent inflammation and the development of hard‐to‐heal wounds [6]. In this context, there is a critical need for therapeutic strategies that combine effective antimicrobial activity with the active promotion of tissue regeneration.

In recent years, innovative treatment alternatives based on polymeric nanomaterials, such as nanofibers, nanoparticles, nanocomposites, and nanohydrogels, have emerged [7]. Topical formulations enriched with natural bioactive compounds, particularly essential oils (EOs), have been investigated as potential approaches for wound management, including both infected and noninfected experimental models; however, most of the available evidence is derived from in vitro studies and animal models [8, 9]. Some EOs also exhibit activity against bacterial biofilms; nevertheless, these effects depend on the type of oil, concentration, and delivery system, and remain limited in in vivo models [10, 11].

Among the promising options, peppermint (*Mentha piperita*), widely used in the form of EO, leaves, extracts, and infusions, deserves particular attention [12]. Peppermint essential oil (PEO) is frequently applied topically due to its anti‐inflammatory, wound healing, antipruritic, astringent, rubefacient, and antiseptic properties, and has also demonstrated activity against viruses, fungi, and bacteria [13, 14].

PEO has additionally been incorporated into topical formulations evaluated in preclinical wound‐healing and infection‐control studies, which report potential benefits such as accelerated wound closure; reduced bacterial load, inflammation, and edema; and enhanced fibroblast migration, collagen synthesis, re‐epithelialization, and modulation of inflammatory cytokines [13].

Although PEO has shown significant potential in the topical treatment of cutaneous infections, no comprehensive synthesis of the scientific literature is currently available to map all tested topical formulations published between 2010 and June 13, 2025, the date on which our search was completed. Therefore, the objective of this scoping review was to systematically identify the formulation of vehicles and concentrations employed, the experimental models adopted (in vitro, ex vivo, and in vivo), and the efficacy parameters assessed. This approach enables the identification of key methodological and knowledge gaps in the field, thereby guiding future research and supporting the development of safer and more effective topical formulations containing PEO.

## 2 Materials and Methods

### 2.1 Search Strategy

**FIGURE 1:**
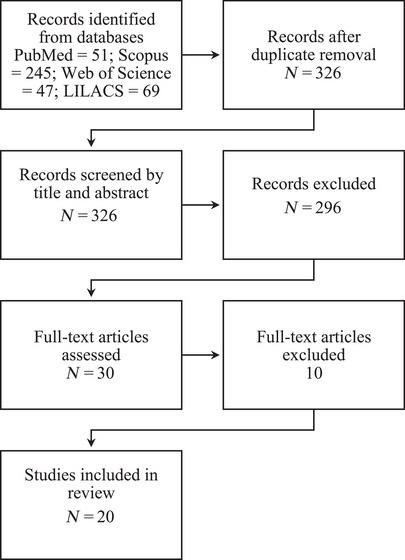
PRISMA flow diagram representing the process of identification, screening, eligibility, and inclusion of studies selected for the review. Adapted from Page et al. [16].

This scoping review was conducted in accordance with the Preferred Reporting Items for Systematic Reviews and Meta‐Analyses extension for Scoping Reviews (PRISMA‐ScR) guidelines [15] (Figure 1). Compliance with the 22 items recommended by the PRISMA‐ScR guidelines was verified (see Table S2 for details). A systematic search was performed in the electronic databases PubMed, Scopus, Web of Science, and LILACS on June 13, 2025.

Eligible studies included original research articles evaluating topical formulations containing PEO (*M. piperita*) for the treatment of infected wounds, published between 2010 and June 13, 2025, in English or Portuguese. This time frame was selected to capture research employing contemporary formulation approaches and recent experimental models. Search strategies were individually adapted for each database and are detailed in Table S1.

This review was prospectively registered in the Open Science Framework (OSF), available at https://osf.io/6jxuw/.

### 2.2 Eligibility Criteria

The eligibility criteria were defined according to the population–concept–context (PCC) framework recommended for scoping reviews:

- *Population (P)*: Studies employing cutaneous infection models, including in vitro, ex vivo, and in vivo in animal models, involving bacteria associated with skin infections;
- *Concept (C)*: Topical formulations containing PEO, either alone or in combination with other components, including creams, gels, ointments, lotions, nanoemulsions, and hydrogels;
- *Context (C)*: Treatment of infected or experimentally induced skin wounds, including sterile wound‐healing models when relevant to the evaluation of topical repair mechanisms.

Although the primary focus of this scoping review was on formulations based on PEO, studies employing extracts or multicomponent systems were also included when *M. piperita* constituted a key active component and when the outcomes were relevant to topical wound healing or antimicrobial performance.

### 2.3 Inclusion and Exclusion

To ensure methodological transparency and reproducibility, inclusion and exclusion criteria were predefined according to the study protocol. Table 1 summarizes the criteria adopted for the selection of studies included in this review.

**TABLE 1:** Inclusion and exclusion criteria adopted in the review.

Category	Inclusion	Exclusion
Period	Publications between January 1, 2010 and June 13, 2025	Publications outside the defined period
Language	English or Portuguese	Other languages
Study type	Original experimental studies (in vitro, ex vivo, in vivo) and case reports testing topical formulations with peppermint essential oil (*Mentha piperita*) for skin infections	Reviews (systematic, narrative, scoping) without original data; conference abstracts without full‐text articles
Formulation	(Implicit in the study type)	Non‐topical formulations (oral, injectable, etc.) or essential oils other than *M. piperita* without direct comparison
Availability	Full‐text accessible articles	Articles without access to the full text

### 2.4 Sources of Information and Search Strategies

The overall search strategy was structured around the combination of three main descriptor blocks: (1) Plant of interest: *M. piperita* OR peppermint oil; (2) clinical condition: wound, healing, skin, cutaneous, lesion, injury, ulcer, inflammation; (3) route of application: topical, formulation, treatment, cream, gel, nanoemulsion, ointment.

Studies published between 2010 and June 13, 2025, in English or Portuguese, were considered for inclusion if they evaluated topical formulations containing PEO for the treatment of infected or experimentally induced skin wounds. The search strategy was adapted to the syntax and specific features of each database (PubMed/MEDLINE, Scopus, LILACS, and Web of Science) to maximize sensibility and relevance in line with the study objectives.

### 2.5 Data Extraction

For data extraction, references retrieved from the four databases (PubMed, Scopus, Web of Science, and LILACS), were imported into the Zotero reference manager. Duplicate records were identified and removed automatically using the “Duplicates” function, which considers title, authorship, and year of publication. The initial total of 412 records was reduced to 326 unique articles, which were subsequently screened by title and abstract using the Rayyan platform.

During the initial screening phase, most records were excluded for recurrent reasons, including: absence of PEO (*M. piperita*) as the intervention; lack of a topical formulation; studies not involving skin, wounds, or cutaneous infection models; absence of antimicrobial or wound‐healing assessments; non‐original articles (e.g., reviews, editorials, or letters); absence of an experimental model (in vitro, ex vivo, or in vivo); or studies in which the EO was not the primary focus of the investigation. Following this stage, 30 articles were selected for full‐text assessment.

Among these, 10 full‐text articles were excluded for the following reasons: use of nonrelevant cutaneous or wound‐healing models (*n* = 5; e.g., dermatitis, pruritus, fungal or algal infections, or inflammatory models without a wound context); absence of isolated PEO in a topical formulation (*n* = 3); and insufficient methodological detail (*n* = 2).

For each included study, the following parameters data were extracted: type of topical formulation; concentration of PEO; experimental model (in vitro, ex vivo, or in vivo); treatment duration, when applicable; antimicrobial and antibiofilm outcomes; wound‐healing effects; and cytotoxicity or safety assessments.

## 3 Results and Discussion

The database search retrieved 412 records (PubMed = 51; Scopus = 245; Web of Science = 47; LILACS = 69). After duplicate removal, 326 articles remained for screening. These records were assessed based on titles and abstracts, resulting in the exclusion of 296 studies that did not meet the eligibility criteria. Consequently, 30 articles were selected for full‐text evaluation. Following a detailed assessment, 10 studies were excluded, primarily because they did not investigate topical formulations containing PEO, evaluated different plant species, or failed to provide data relevant to the scope of this review. Ultimately, 20 studies were included in the final analysis (Table 2).

**TABLE 2:** Compilation of in vitro, in vivo, and ex vivo studies evaluating topical formulations containing *Mentha piperita* essential oil.

References	Type of study	Main assays	Main findings
[17]	In vivo	Wound area, contraction rate, hematological parameters, and histology (cell infiltration, inflammation, tissue regeneration)	*M. piperita* promoted complete wound healing in 18 days; *Cymbopogon citratus* in 20 days; normal rats healed in 12 days; untreated diabetic rats remained with incomplete healing. Both oils reduced inflammation and improved skin regeneration, being more effective than the antibiotic control
[18]	In vitro	Physicochemical parameters, biofilm penetration, antimicrobial activity, comparison between free and encapsulated oil, cytotoxicity, and cell proliferation	Pickering capsules containing PEO, especially CP‐Cap (with cinnamaldehyde), exhibited strong antibiofilm activity, reduced MRSA viability, and stimulated fibroblast proliferation without toxicity, unlike free oil, which was cytotoxic
[19]	In vitro and in vivo	Size, PDI, zeta potential, encapsulation efficiency, drug loading, MIC, MBC, histology, and expression of FGF‐2 and EGF	PEO‐NLC showed antimicrobial activity and reduced bacterial load; stimulated fibroblasts, collagen deposition, neovascularization, and accelerated epithelialization. It also increased FGF‐2 and EGF expression, accelerating healing between Days 7 and 14
[20]	In vivo	Wound contraction, bacterial count in granulation tissue, pathological observations, and target gene mRNA expression	PEO reduced bacterial load and edema, stimulated fibroblasts, collagen deposition, and epithelialization. Concentrations of 4% and 8% enhanced wound contraction, with efficacy comparable to mupirocin. Higher doses promoted significantly greater re‐epithelialization
[21]	In vivo	Wound area, contraction rate, hydroxyproline, hexosamine, and uronic acid levels, plus histopathology of total cells, vessels, fibroblasts, and fibrocytes	The *M. piperita* ointment significantly reduced wound area, increased contraction, elevated biochemical markers of collagen synthesis, and improved histological parameters, showing effects comparable or superior to tetracycline in some aspects
[22]	In vivo	Wound area, healing percentage, fibroblasts, vessels, inflammatory cells, collagen fibers, and TGF‐β gene expression	The essence + Vaseline group achieved 99.7% wound healing in 14 days; essence alone 74.6%; Vaseline alone 67%; control only 52%. Treated groups showed increased fibroblasts, vessels, and collagen fibers, and reduced inflammatory cells. RT‐PCR revealed increased TGF‐β expression in treated groups
[23]	In vivo	Wound contraction, fasting glucose, biochemical profile (renal, hepatic, lipid, protein), gene expression (WNT4, MMP9, TGF‐β1, 5srRNA, CTNNB1), and skin histology	In diabetic rats, healing was slow, but hydrogel with PEO and silver nanoparticles showed the best performance, achieving complete closure in 16 days. It also reduced fasting blood glucose, modulated regeneration‐related genes, and improved histological parameters, with enhanced collagen and re‐epithelialization
[24]	In vitro and ex vivo	Physicochemical, thermal, mechanical, morphological, surface, diffusion, and chemical characterization of emulsions and electrospun matrices to determine stability, functionality, and oil release	Electrospun PVA/chitosan matrices incorporating peppermint essential oil emulsion showed mechanical stability, chemical interactions, skin diffusion capacity, and potential for topical or transdermal applications
[25]	In vitro	Antimicrobial activity (MIC, MBC, agar diffusion); chemical and antioxidant characterization; morphology; cytotoxicity	The MICs were in the range of 5.7–9.4 L/mL, and MBCs 9.4–25.0 µL/mL with similar results against *Staphylococcus aureus* depending on *Mentha* species. Nanofibers with PEO exhibited antimicrobial effect without affecting surrounding microbiota
[26]	In vitro and in vivo	Physicochemical characterization; antibacterial activity (agar diffusion, MIC, MBC, time–kill curves); in vivo wound contraction, bacterial load, cell viability, histology; and gene expression of TNF‐α, caspase‐3, Bcl‐2, cyclin D1, and FGF‐2	Au/γ‐AlOOH‐NC and Au/γ‐AlOOH/Ctn‐NC showed strong antimicrobial activity, comparable to mupirocin and vancomycin, accelerating wound contraction, reducing edema and bacterial load, and stimulating fibroblasts, collagen deposition, and epithelialization. They also modulated inflammatory and proliferative markers, favoring the proliferative phase of healing
[27]	In vitro	Droplet size (TEM), morphology (SEM), FT‐IR, viscosity/conductivity, tensile/elongation strength; antibacterial activity, protein leakage, biofilm inhibition, and microtoxicity	The PCL‐4 formulation demonstrated potent antimicrobial and antibiofilm activity, with complete inhibition at 200 µg/mL and MIC/MBC values between 25/50 and 100/125 µg/mL. Encapsulation of the essential oil nanoemulsion enhanced PCL activity, outperforming both isolated nanoemulsion and pure polymer
[28]	In vivo and in vitro	Physicochemical properties, fluid absorption, biodegradation, hemolysis, cell adhesion, migration, proliferation, histology, immunohistochemistry, collagen maturation, bacterial colonization, and hematological parameters	All treatments favored healing, but 4% PEO was the most effective and safe concentration. Conversely, 8% caused irritation and inflammation in animals. In vivo, the Pu/Gelatin/PEO (4%) formulation showed superior healing performance and adhesive stability compared to others
[29]	In vitro	pH, viscosity, spreadability, extrudability, drug content, in vitro release, washability, and antimicrobial activity	The gels displayed adequate physicochemical profiles and dose‐dependent antimicrobial activity. PEO was most effective against *Pseudomonas aeruginosa* and showed good inhibition of *Escherichia coli* and *Aspergillus tubingensis*, but weaker effects against *S. aureus* and *Bacillus subtilis*
[30]	In vivo	Wound healing rate; histopathology; re‐epithelialization score; collagen deposition; and inflammation status	The *M. piperita* + clinoptilolite group exhibited significantly increased fibroblasts and vessels, along with reduced inflammatory cells compared to other groups
[31]	In vitro	Morphology (SEM), chemical composition (Raman, GC–MS), wettability, in vitro degradation, menthol content, antibacterial activity, and cell viability	PCL fibers with 1.5%–6% PEP showed reduced diameter (∼1.0 µm), effective antibacterial activity against *S. aureus* and *E. coli* (especially at 6%), and no cytotoxicity to human fibroblasts, indicating potential as antibiotic‐free wound dressings
[32]	In vitro and in vivo	Antimicrobial activity and tissue histomorphology: inflammation, epithelialization, neovascularization, fibroblast proliferation, collagen deposition	Nanoemulsions reduced IL‐1β, TGF‐β1, and MDA, increased GSH, and improved epithelialization, collagen deposition, and angiogenesis, particularly in the lavender + frankincense + peppermint combination. Blends were more effective, promoting faster healing, reduced scarring, and antimicrobial activity against *S. aureus* and *E. coli*
[33]	In vitro and in vivo	Droplet size, PDI, zeta potential, particle morphology, physicochemical aspects of the gel, skin safety, and antimicrobial activity	Microwave‐assisted spherical nanoemulsions produced stable, homogeneous nanoemulgels, with no irritation in 30 volunteers. Antimicrobial activity was dose‐dependent, with HN5 (peppermint:myrtle 1:1) showing the greatest effect against *S. aureus* and *E. coli*
[34]	In vitro and in vivo	Antioxidant, antiglycation, antimicrobial activity; mass spectrometry analysis; dermal toxicity; diabetes induction; wound induction; wound healing and histopathology	Hydrocolloid gel with AgNPs from *M. piperita* showed enhanced antioxidant, antiglycation, and antimicrobial activity, promoting 100% wound closure in 20 days and accelerated re‐epithelialization
[35]	In vitro	Particle size, distribution uniformity, surface potential, morphology (TEM), antimicrobial activity, and biofilm inhibition	Lipid nanocapsules (LNCs) formulated with peppermint oil as the oily core and loaded with levofloxacin (LFX‐LNCs) showed antimicrobial activity against *E. coli*, *S. aureus*, and for *P. aeruginosa*, and improved biofilm inhibition
[36]	In vitro	Droplet size, zeta potential, pH, viscosity, spreadability, accelerated stability, in vitro permeation, and antimicrobial activity	The NC4 nanoemulsion was stable, translucent, and showed 97.1% permeation within 10 h, outperforming conventional cream (62.4%). It demonstrated strong antimicrobial activity against *S. aureus* and *E. coli* and remained stable for 6 months without phase separation

*Note*: The table summarizes types of study, assays performed, and main findings related to antimicrobial activity and wound healing efficacy (2010–June 13, 2025).

Abbreviations: 5srRNA, 5 small ribosomal RNA; AgNPs, silver nanoparticles; Au, gold; Au/γ‐AlOOH/Ctn‐NC, gold/gamma‐aluminum oxyhydroxide/chitosan nanocomposite; Bcl‐2, B‐cell lymphoma 2; CMC, carboxymethylcellulose; CP‐Cap, capsules containing 5% v/v of cinnamaldehyde dissolved in peppermint oil; CTNNB1, catenin beta‐1; EGF, epidermal growth factor; ex vivo, assay using excised tissue samples; FGF‐2, fibroblast growth factor‐2; FT‐IR: Fourier transform infrared spectroscopy; GSH, glutathione; HN5, hand nanoemulgel 5 (fifth formulation in the tested series); IL‐1β, interleukin‐1 beta; in vitro, assay under controlled laboratory conditions; in vivo, assay performed in living organisms; MBC, minimum bactericidal concentration; MDA, malondialdehyde; MIC, minimum inhibitory concentration; MMP9, matrix metalloproteinase‐9; NC4, nanocream 4 (the optimum nanocream formulation); NLC, nanostructured lipid carriers; PCL, poly(ε‐caprolactone); PDI, polydispersity index; PEO, peppermint essential oil; PEO‐NLC, peppermint essential oil loaded into nanostructured lipid carriers; PEP, peppermint oil; PVA, polyvinyl alcohol; RT‐PCR, reverse transcription polymerase chain reaction; SEM, scanning electron microscopy; TEM, transmission electron microscopy; TGF‐β, transforming growth factor‐beta; TNF‐α, tumor necrosis factor alpha; WNT4, wingless‐type MMTV integration site family member 4; ZOI, zone of inhibition; γ‐AlOOH‐NC, gamma‐aluminum oxyhydroxide nanocomposite.

These findings highlight both the limited number of investigations specifically focused on topical formulations containing PEO and the heterogeneity of experimental approaches employed. A substantial proportion of studies were excluded due to the use of different *Mentha* species, the evaluation of peppermint oil exclusively in combination with other active agents rather than as a primary component, or insufficient methodological and outcome‐related data to address the objective of this review.

### 3.1 Study Design of Included Articles

Although case reports were considered eligible according to the predefined criteria, none of them met the inclusion requirements following full‐text assessment. Of the 20 included studies, the vast majority consisted of preclinical investigations. Seven studies (35%) were conducted exclusively in vitro, primarily focusing on physicochemical characterization of the formulations and the assessment of antimicrobial activity. Six studies (30%) combined in vitro and in vivo assays, enabling the evaluation of both laboratory‐based parameters and biological effects in animal models. An additional six studies (30%) were conducted solely in in vivo models, emphasizing wound‐healing outcomes and antimicrobial efficacy under experimentally induced infection conditions.

**FIGURE 2:**
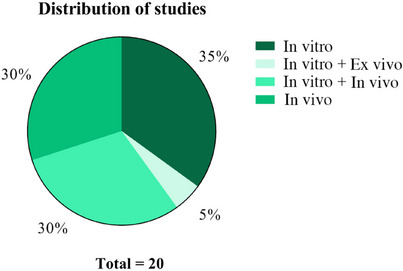
Distribution of study designs among the included articles. The figure illustrates the proportion of studies according to the experimental model employed, including in vitro, in vivo, combined in vitro/in vivo, and combined in vitro/ex vivo approaches.

Notably, the included studies exhibited substantial heterogeneity in experimental design, encompassing both infected and noninfected wound models, as well as formulations in which PEO was evaluated either as the primary active component or as part of multicomponent systems. Only one study (5%) employed a combined in vitro and ex vivo approach, using skin as a complementary biological model. No clinical trials were identified, underscoring the early and exploratory stage of research on PEO‐based topical formulations of cutaneous infections. The distribution of study designs is summarized in Figure 2, which illustrates the predominance of preclinical evidence and the absence of clinical investigations.

### 3.2 Phytochemical Profiles

*M. piperita* is one of the most widely used herbs worldwide, with a long history of safe use in medicinal preparations [37]. Scientific studies have reported its biological effects, including antioxidant, antimicrobial, antiviral, anti‐inflammatory, biopesticidal, larvicidal, anticancer, radioprotective, genotoxic, and antidiabetic activities [38]. The species is a rich source of phytoconstituents of diverse chemical natures and can be considered an important source of bioactive substances for health improvement and for the development of medicinal products used in complementary therapies for various conditions, particularly those related to oxidative stress, inflammation, and moderate infections [39].

Its broad spectrum of biological activity is attributed to its chemical composition, predominantly menthol, menthone, neomenthol, and isomenthone, which exhibit well‐characterized anti‐inflammatory, antibacterial, antiviral, antifungal, immunomodulatory, antitumoral, neuroprotective, antifatigue, and antioxidant activities [40]. Essential oils (EOs) are complex mixtures composed of hundreds of constituents, whose composition may vary substantially depending on factors such as the extraction method used and the geographical origin of the plant material [41].

Several studies have demonstrated that the variations in the chemical profiles of PEO are influenced by both environmental and genetic factors. Telci et al. [42] reported that mint clones cultivated in different regions exhibited distinct menthol and menthone contents, which were attributed to differences in climatic conditions and soil composition. In *Mentha pulegium*, Laftouhi et al. [43] showed that variations in temperature and precipitation significantly affected both the yield and the chemical composition of the EO. Furthermore, Hubert‐Schöler et al. [44] demonstrated that different genotypes respond differently to light intensity (shading), resulting in changes in the relative proportions of major monoterpenes.

Studies conducted in different geographical locations have also revealed considerable variation in EO composition. Chemical analysis performed in Pakistan by Fazal et al. [45] showed that the main EO constituents were carvone, linalool, hotrienol, menthol, isopulegone, furanone, piperitone, and thymol. In contrast, a study conducted in Prešov, Slovakia, identified menthol (70.08%) and menthone (14.49%) as the major components, followed by limonene (4.32%), menthyl acetate (3.76%), and β‐caryophyllene (2.96%) [46]. In Morocco, the chemical analysis of the EO revealed menthol (46.32%), menthofuran (13.18%), menthyl acetate (12.10%), menthone (7.42%), and 1,8‐cineole (6.06%) as the predominant constituents [47].

Among the constituents of *M. piperita* EO, menthol and menthone stand out as the primary bioactive compounds and are widely recognized as mainly responsible for its antimicrobial and antibiofilm effects. As demonstrated by Trombetta et al. [48], menthol can disrupt the lipid fraction of the bacterial plasma membrane, compromising membrane integrity and increasing cellular permeability. This mechanism underlies the significant antibacterial activity of menthol against pathogenic microorganisms and supports its potential use as an alternative or adjunct to conventional antibiotics [49]. Even at subinhibitory concentrations, menthol has been reported to inhibit quorum‐sensing mechanisms in Gram‐negative bacteria, making it a promising strategy for attenuating bacterial virulence rather than acting solely as a bactericidal agent [50]. Furthermore, both menthol and menthone are capable of inserting into cellular membranes, inducing changes in permeability and disruption of the lipid bilayer, thereby interfering with essential cellular processes and impairing bacterial adhesion and biofilm maturation [51].

Variation in EO composition, as well as differences in incorporation systems, can directly influence the biological activities observed. Among the studies included in this review (*n* = 20), only two (10%) reported the chemical composition of the PEO used. The remaining studies primarily focused on the preparation, characterization, and biological evaluation of the formulations, rather than on a detailed phytochemical analysis of the EO itself.

### 3.3 Developed Formulations

The formulations developed in the included studies exhibited considerable diversity, ranging from conventional vehicles to nanostructured systems. In earlier years (2013–2015), simpler formulations predominated, such as classical ointment bases and encapsulated emulsions [17, 18]. From 2019 onward, although conventional formulations such as ointments continued to be reported [20, 21], nanostructured lipid carriers were also introduced [19], reflecting the growing influence of nanotechnology in topical drug delivery.

In 2022, hybrid formulations emerged, including hydrogel films and combinations of pure EO with basic vehicles such as petrolatum [22, 23]. More recently, between 2023 and 2025, studies explored electrospun systems, including nanofibers composed of poly(ε‐caprolactone), gelatin, and polyvinyl alcohol (PVA) combined with chitosan. Additional approaches included nanoemulsions, nanogels, nanocreams, lipid nanocapsules, and formulations combined with metallic nanoparticles (Table 3). In these multicomponent systems, the observed biological effects cannot be attributed exclusively to PEO, but rather reflect the combined contribution of the carrier system and additional active components. These advances illustrate a clear shift toward increasingly sophisticated delivery platforms and highlight the growing technological refinement in the development of PEO‐based topical products.

**TABLE 3:** Topical formulations containing *Mentha piperita*‐derived products, number of publications per formulation, concentrations, and treatment duration, when applicable.

Formulation	*N*	Concentration	Treatment duration
Conventional vehicles			
Ointment	4 (20%)	Two articles did not report concentration; 2%, 4%, and 8% (v/v); 3% (w/w) extract in Eucerin	12, 14, 20, or 30 days
Vaseline	1 (5%)	Not reported	14 days
Nanocream (nanoemulsion + cream base)	1 (5%)	594 mg PEO per 100 g cream (0.594% w/w)	No applicable
Capsule systems			
Pickering capsule (pickering, PVA, and chitosan)	1 (5%)	Not reported	No applicable
Lipid nanocapsules (LNCs)	1 (5%)	Not reported	No applicable
Encapsulated emulsion—PVA and chitosan	1 (5%)	7.5% (v/v)	No applicable
Nanostructured systems			
Nanoemulsion incorporated into spray	1 (5%)	10% (v/v)	15 days
Nanoemulgel	1 (5%)	5% (HN1) and 10% (HN2) (v/v)	No applicable
Nanostructured lipid carriers	1 (5%)	Not reported	14 days
Solid formulations/films for sustained release			
Hydrogel	1 (5%)	Not reported	30 days
PCL—poly(ε‐caprolactone) dermal patch	1 (5%)	Not reported	29 days
Pullulan dermal patch	1 (5%)	2%, 4%, and 8%(v/v)	21 days
Topical gel with carbopol	2 (10%)	5 mL and 2 mL/0.5 mg/g (w/w)	No applicable or 29 days
Hybrid structures composed of electrospun poly(ε‐caprolactone) (PCL)	1 (5%)	1.5%, 3%, and 6% (v/v)	No applicable
Gelatin nanofibers	1 (5%)	2% and 20% (w/w)	No applicable
Clinoptilolite	1 (5%)	Not reported	14 days

*Note*: Treatment duration refers exclusively to therapeutic in vivo studies involving repeated application over time, in vitro, ex vivo, and clinical skin irritation assays involved single‐ or short‐term exposure and therefore do not represent treatment duration; in these cases, treatment time was reported as not applicable.

The analyzed literature reveals a wide diversity of pharmaceutical vehicles used for PEO incorporation, reflecting both the interest in improving compound stability and the intention to enhance its biological effects. Umasankar et al. [17] evaluated PEO‐based ointments in diabetic wound healing within 20 days, accompanied by histological and biochemical improvements, although the concentration of EO was not clearly specified. These findings suggest that traditional semisolid vehicles can serve as viable matrices for topical delivery, despite the lack of standardization regarding dose limits.

Nanoparticles loaded with EO may offer several advantages, including synergistic antimicrobial activity, high loading capacity, increased solubility, reduced volatility, improved chemical stability, and shelf life of EO and its constituents [52]. However, in the absence of direct comparative experimental designs, the available evidence does not allow the conclusion that nanoformulations are intrinsically more potent than conventional formulations.

Ghodrati et al. [19] investigated lipid nanocarriers for PEO encapsulation and obtained particles with good physicochemical stability and in vitro antimicrobial activity against *Staphylococcus aureus* and *E. coli*. In in vivo models, these formulations reduced bacterial load and accelerated wound contraction, indicating that nanostructured systems may be advantageous both for protecting volatile compounds and for improving skin penetration. Similar results were reported by Duncan et al. [18], who employed nanoparticle‐stabilized capsules (Pickering emulsions), further highlighting the potential of innovative vehicles to enhance antimicrobial efficacy and enable controlled local release.

Incorporation of EO into nanostructured systems, such as nanoemulsions, nanocreams, lipid carriers, and metallic nanoparticles, was frequently associated with enhanced antimicrobial performance within individual studies. However, in many cases, these effects resulted from combined formulations involving PEO together with metallic nanoparticles, antibiotics, or other EOs, rather than PEO alone. This suggests synergistic interactions and improved bioavailability as contributing factors. As discussed by Santos et al. [53], reduced droplet size in nanoemulsions may enhance interactions between EO components and bacterial cell membranes, which has been proposed as one of the mechanisms underlying their antimicrobial activity. Nevertheless, due to the lack of direct comparative studies, these mechanistic considerations should be considered with caution.

The efficacy of PEO in simple vehicles was also confirmed in infected models. Modarresi et al. [20] tested ointments containing EO in rats with wounds contaminated by *S. aureus* and *Pseudomonas aeruginosa*, observing a significant reduction in bacterial load and an increased rate of wound contraction. These findings suggest that conventional topical formulations, when properly standardized, may achieve therapeutic outcomes comparable to those of more complex systems, reinforcing the clinical potential of PEO. Complementarily, Zangeneh et al. [21], demonstrated that incorporation of an aqueous extract of *M. piperita* into an ointment with an Eucerin base promoted wound healing in a noninfected model, associated with modulation of inflammatory parameters, although without direct antimicrobial evaluation. As this study employed an aqueous extract rather than isolated EO and did not involve microbial challenge, the observed effects are more appropriately interpreted as wound‐healing modulation rather than direct antimicrobial activity of PEO.

Overall, the evidence indicates that PEO exhibits substantial potential when incorporated into topical formulations, combining significant antimicrobial activity with anti‐inflammatory and wound‐healing properties. The analyzed studies demonstrated that formulation strategies range from traditional ointments to advanced nanostructured systems, each presenting specific advantages and limitations. Regardless of the technological complexity of the delivery system, the presence of PEO was consistently associated with favorable wound‐healing outcomes, supporting its potential as a therapeutic agent for topical applications.

### 3.4 Concentrations Used

The concentration of PEO employed in the analyzed formulations varied widely among the included studies (Table 3). The lowest concentrations, around 0.5%, were generally associated with controlled‐release systems or formulations combined with metallic nanoparticles [23, 34]. In contrast, some studies reported substantially higher concentrations, reaching up to 20% (w/w) in gelatin‐based matrices [25]. Intermediate concentrations, ranging from 1.5% to 8%, were most frequently applied, particularly in lipid nanocarriers, nanocreams, and topical gel formulations.

In addition, some studies reported dosages in terms of the amount applied (mg/day), whereas others expressed concentrations as weight per volume (w/v), µg/mL, mg/mL, or other units. This heterogeneity complicates standardization and direct comparison of results. Although concentration standardization would ideally facilitate cross‐study comparisons, it was not feasible in the present review due to substantial differences in how *M. piperita*‐derived products were quantified. Achieving standardization would have required assumptions regarding formulation density, extraction yield, vehicle composition, or mass–volume equivalence, parameters that were not consistently reported in the studies.

In several studies, concentrations were expressed using fundamentally different units, while in others, only the applied dose was reported, or no concentration data were provided. Notably, 9 of the 20 included studies (45%) did not report concentration values for *M. piperita*‐derived products, thereby limiting quantitative comparison among formulations. As a result, numerical standardization would necessitate assumptions regarding formulation density, extraction yield, or vehicle composition, which were not consistently available. Therefore, concentrations were retained as reported in the primary studies to preserve methodological accuracy. Overall, this variability indicates that, although there is broad agreement regarding the antimicrobial potential of PEO, there is still no consensus on the optimal concentration for topical use, an aspect that should also be evaluated in relation to skin toxicity [54].

### 3.5 Tested Microorganisms and Antimicrobial Activity

*S. aureus* was the most frequently tested species, including both susceptible and resistant strains, reflecting its clinical relevance in cutaneous infections. Another commonly tested pathogen was *E. coli*, representing the most frequent Gram‐negative model (Table 4). This predominance is explained by the fact that *E. coli* and *S. aureus* are widely recognized as representative Gram‐negative and Gram‐positive bacterial pathogens, respectively [55].

**TABLE 4:** Frequency of microorganisms evaluated in the included studies.

Microorganism	*n*	%
*Staphylococcus aureus*	12	60
*Escherichia coli*	8	40
*Pseudomonas aeruginosa*	7	35
*Staphylococcus epidermidis*	2	10
*Listeria monocytogenes*	2	10
*Bacillus subtilis*	2	10
*Candida albicans*	2	10
*Proteus mirabilis*	1	5
*Enterobacter cloacae* complex	1	5
*Streptococcus pneumoniae*	1	5
*Streptococcus pyogenes*	1	5
*Salmonella typhimurium*	1	5
*Acinetobacter baumannii*	1	5
*Cutibacterium acnes*	1	5
*Micrococcus luteus*	1	5
*Bacillus anthracis*	1	5
*Aspergillus fumigatus*	1	5
*Aspergillus niger*	1	5
*Aspergillus tubingensis*	1	5
No experimental bacterial inoculation	6	30

*Note*: The table summarizes the microorganisms investigated across the included studies. Frequencies represent the number of times each microorganism was evaluated, considering all reported experimental assessments. Studies without experimental microbial inoculation were included.

Some studies did not include bacterial inoculation in animal models, instead focusing on physicochemical characterization or sterile wound‐healing approaches (*n* = 6). Despite the frequent use of reference microorganisms, considerable variability was observed in the microbiological models employed. In addition, bacterial species investigated included *Streptococcus pyogenes*, *Staphylococcus epidermidis*, *Cutibacterium acnes*, *Acinetobacter baumannii*, and *Listeria monocytogenes*, as well as fungal species such as *Candida albicans*, *Aspergillus niger*, and *Aspergillus tubingensis*.

For antimicrobial activity assessment, the included studies employed a wide range of methodologies, including determination of minimum inhibitory concentration (MIC), minimum bactericidal concentration (MBC), and zone of inhibition (ZOI) assays. However, the lack of methodological uniformity across studies, encompassing differences in initial tested concentration ranges, formulation types, antimicrobial endpoints, and experimental protocols, precludes direct comparison of antimicrobial susceptibility among microorganisms. In this context, although *S. aureus, E. coli*, and *P. aeruginosa* were the most frequently investigated species, it is not methodologically appropriate, within the scope of the present review, to infer their relative susceptibility to PEO.

### 3.6 Treatment Duration

Regarding treatment duration, substantial variability was observed across the included studies. Exclusively in vitro experiments were generally limited to single‐point analyses without a defined exposure period, whereas in vivo models reported intervention durations ranging from 12 to 29 days, most commonly involving once‐daily topical application. In some studies, only the applied amount (mg or g per day) was specified, without clear information on the total duration of treatment, which limits direct comparison of experimental protocols.

This lack of uniformity reflects both the absence of standardized application regimens and the need to tailor experimental designs to different formulations and wound models. Importantly, such variability is not unique to the present review but represents a well‐recognized limitation in preclinical wound‐healing research. To address this gap, the Wound Reporting in Animal and Human Preclinical Studies (WRAHPS) Guidelines were developed to promote standardized reporting of key parameters, including dose, route, frequency, and duration of treatment [56]. Similarly, the ARRIVE 2.0 guidelines emphasize detailed reporting of intervention regimens in in vivo studies. In line with these concerns, previous systematic reviews in wound research, including those evaluating topical herbal products, have reported substantial heterogeneity in treatment protocols and outcome measures, which continues to limit cross‐study comparability [57].

When the included studies are examined in light of the recommendations proposed by the WRAHPS and ARRIVE 2.0 guidelines, it becomes evident that gaps in standardization are accompanied by recurrent reporting deficiencies. Although most studies reported the general treatment period or daily application of the formulations, critical details regarding dosage, application frequency, and concentration were frequently incomplete or inconsistently described, particularly in in vivo models.

In several cases, only general information on daily application was provided, without a clear specification of application frequency or the effective concentration of the active compound. Considering the cited guidelines, which identify detailed reporting of these parameters as essential for experimental reproducibility and translational interpretation, the inconsistency observed across the included studies represents a relevant methodological limitation of the current literature.

### 3.7 Safety and Toxicity

Overall, eight of the included studies (40%) evaluated toxicity using in vitro, in vivo, or clinical approaches. Among these, two studies (10%) reported low cytotoxicity in in vitro assays, one study (5%) reported low toxicity in an in vivo model, and five studies (25%) reported no toxic effects. Notably, the studies reporting higher toxicity were also those employing higher concentrations of *M. piperita*, suggesting that the observed adverse effects are more likely related to the concentration used rather than to the intrinsic properties of the product itself (Table 5).

**TABLE 5:** Safety and toxicity outcomes of peppermint essential oil‐based topical formulations.

References	Formulation type	*Mentha piperita* product	Reported concentration	Method	Outcome
[31]	Hierarchical PCL–gelatin nanofiber scaffold	Essential oil	Not reported	In vitro cytotoxicity assay	No cytotoxicity observed
[34]	Hydrocolloid film with AgNPs	Extract/essential oil	Not reported	In vivo dermal toxicity	No cytotoxicity observed
[25]	Gelatin nanofibers	Essential oil	20% (w/w)	In vitro cytotoxicity assay	Low cytotoxicity; concentration‐dependent effects
[26]	Au/γ‐AlOOH nanocomposite (ointment)	Extract	Not reported	In vitro cytotoxicity assay	No cytotoxicity observed
[18]	Nanoparticle‐stabilized capsules (Pickering emulsion)	Essential oil	Not reported	In vitro cytotoxicity assay	No cytotoxicity observed
[27]	Polycaprolactone (PCL) electrospun nanofibers loaded with peppermint oil nanoemulsion	Essential oil	Not reported	In vitro toxicity assay	Low cytotoxicity
[28]	Pullulan‐based microporous scaffolds combined with gelatin, alginate, chitosan or CMC	Essential oil	2%, 4%, and 8% (v/v)	In vitro cytotoxicity assay and in vivo dermal toxicity	No cytotoxicity observed biocompatibility and in vivo safety, whereas 8% caused skin irritation
[33]	Nanoemulgel for skin application	Essential oil	5% and 10% (w/w)	Human skin irritation test (30 volunteers)	No skin irritation or adverse reactions reported

*Note*: The table summarizes studies reporting any form of safety or toxicity assessment using in vitro, in vivo, or clinical models.

Although EOs are generally regarded as safe at low concentrations, evidence indicates that their allergenic or irritant potential increases at higher doses or when inadequately formulated [58]. Consistent with this observation, the present review shows that reports of increased toxicity were restricted to studies using higher concentrations of *M. piperita*, supporting a concentration‐dependent effect rather than an inherent toxicity of the EO.

From a mechanistic perspective, EOs and their volatile constituents can penetrate the skin primarily by disrupting the highly ordered intercellular lipid structure of the stratum corneum, thereby reducing the barrier function of this layer and facilitating the diffusion of compounds into deeper skin regions [59]. While this mechanism is advantageous for enhancing cutaneous permeation, it also increases the exposure of viable epidermal cells to bioactive components of the EO. At elevated concentrations or in the absence of appropriate formulation control, this increased exposure may contribute to irritant reactions, cytotoxicity, or skin sensitization, thereby explaining the dose‐dependent nature of the observed toxicity.

### 3.8 Observed Patterns

Overall, the analyzed studies revealed consistent methodological patterns. A predominance of in vitro assays was observed, indicating that much of the literature remains focused on the initial characterization of formulations and the evaluation of their antimicrobial efficacy under controlled conditions, with limited validation in more complex biological models. The distribution of tested microorganisms, including the predominance of specific bacterial species, is summarized in Table 4.

In addition, a growing trend has been observed, particularly over the past five years, toward incorporating EO into nanostructured systems. This strategy aims to overcome limitations related to volatility and chemical instability while enhancing skin permeation and biological activity. Compared with conventional emulsions, such nanoformulations offer several advantages, as their reduced droplet size provides greater kinetic stability, increased interfacial area, and improved optical transparency [60].

### 3.9 Gaps and Implications

Despite their widespread use, EOs face several challenges in skincare formulations, including instability, volatility, and the risk of skin irritation, which have limited their full therapeutic potential [61]. Although methodological and technological advances have been reported, the analysis of the included studies revealed important gaps that hinder the consolidation of evidence. Substantial methodological heterogeneity was identified, including wide variations in oil concentrations, formulation types, treatment durations, and experimental models, which complicate direct comparison between studies.

Notably, the absence of ex vivo studies using human skin was also evident. Such models may more accurately reflect human skin physiology in terms of structure, cell signaling pathways, metabolic activity, biomarker expression, and absorption profiles [62]. Furthermore, many studies prioritized physicochemical characterization or basic antimicrobial assays without progressing to more complex biological models. These gaps highlight the need for methodological standardization and for research approaches that are more clinically oriented, capable of generating robust and translatable data on the efficacy, safety, and clinical applicability of PEO in cutaneous infections.

The implications of these findings suggest that, although PEO exhibits promising antimicrobial and wound‐healing potential in topical formulations, the current body of literature remains largely exploratory. The increasing incorporation of nanotechnologies represents a consistent strategy to improve oil stability and biological performance, while the investigation of synergistic combinations with conventional drugs opens new therapeutic perspectives [63]. Nevertheless, these advances require validation in well‐designed preclinical and clinical studies to confirm their safety and therapeutic effectiveness.

### 3.10 Limitations of This Study

This scoping review has several limitations that should be considered. First, the literature search was restricted to articles published in English or Portuguese, which may have resulted in the exclusion of relevant studies published in other languages. In addition, although four major databases were consulted (PubMed, Scopus, Web of Science, and LILACS), the review relied exclusively on electronic databases and did not include grey literature sources, such as theses, dissertations, conference proceedings, or patent documents, potentially limiting the identification of emerging or non‐indexed evidence. As the search was conducted on a single date (June 13, 2025), studies published after this date were not included.

Most of the included studies were preclinical, involving in vitro assays and in vivo animal models, with no clinical trials in humans, which limits the direct translation of findings into clinical practice. As expected for predominantly preclinical literature, several methodological limitations were identified, including small sample sizes, lack of randomization or blinding, incomplete reporting of experimental procedures, and inconsistent characterization of PEO composition. These limitations may affect internal validity and restrict cross‐study comparability. In particular, variability in oil concentrations, formulation strategies, treatment duration, and microbiological methods precluded direct comparison of outcomes and prevented any form of quantitative synthesis. Nevertheless, this scoping review provides a comprehensive mapping of topical *M. piperita*‐based formulations and highlights critical methodological gaps that should inform the design and reporting of future studies.

## 4 Conclusion

This scoping review demonstrates that PEO has been investigated in a wide range of topical formulations, including both conventional vehicles and more recent nanostructured systems. The analyzed studies highlight its antimicrobial and wound‐healing potential, particularly against *S. aureus*, the pathogen of greatest clinical relevance in cutaneous infections.

Importantly, the included studies employed both infected wound models and noninfected (sterile) wound‐healing models. In infected models, *M. piperita*‐containing formulations were primarily associated with antimicrobial effects and reductions in bacterial burden, whereas in noninfected models, the reported outcomes mainly reflected modulation of inflammation, tissue repair, and wound closure.

Studies employing nanostructured delivery systems reported favorable outcomes related to formulation stability and topical performance; however, the available evidence remains heterogeneous and is largely limited to preclinical investigations. Incorporation into nanostructured systems was associated with improved stability, enhanced skin permeation, and increased antimicrobial efficacy, particularly when combined with nanoparticles, metallic components, other bioactive compounds, or, in one case, a conventional antibiotic such as levofloxacin.

Notably, most studies relied on in vitro assays and animal models, with substantial methodological variability regarding concentrations, formulation types, treatment durations, and microbiological parameters. This variability, together with the limited number of safety assessments, considerably restricts the translation of experimental findings into clinical practice. Although some studies conducted in vitro cytotoxicity assays and limited skin irritation evaluations, no clinical investigations assessed systemic or long‐term safety.

Therefore, future research should prioritize more rigorous and standardized experimental approaches, including ex vivo human skin models, bacterial biofilm systems, reconstructed human skin equivalents, and, critically, well‐designed clinical trials. Such efforts are essential to consolidate the role of PEO as a therapeutic alternative for the treatment of cutaneous infections.

## Funding

The authors thank the National Council for Scientific and Technological Development (CNPq, grant 307035/2022‐0) and the Coordination for the Improvement of Higher Education Personnel (CAPES, Finance Code 001) for their research support.

## Conflicts of Interest

The authors declare no conflicts of interest.

## Supporting information




**Supporting File**: cbdv70936‐sup‐0001‐SuppMat.docx

## Data Availability

All data generated or analyzed during this study are included in this published article and its supplementary information files. The protocol is available at OSF (https://osf.io/6jxuw/).
