# Supplementary material for: Topical Formulations Containing Mentha piperita for Wound Healing: A Scoping Review
Source: Chem Biodivers. 2026 Jan 31;23(2):e03567. doi: 10.1002/cbdv.202503567 (PMC12860549; doi:10.1002/cbdv.202503567)
Supplement: Supplementary file 1 — Supporting File: cbdv70936‐sup‐0001‐SuppMat.docx [file CBDV-23-e03567-s001.docx]

**Material Supplementary/ Supplementary Tables**

**Supplementary Table S1**. The PRISMA-ScR checklist was completed for this scoping review to ensure compliance with the 22 recommended items and to indicate how they were addressed in the manuscript ^[13]^.

| **Item** | **Description** | **Addressed in the manuscript** | **Section/Page** |
| --- | --- | --- | --- |
| 1 | Title identifies the report as a scoping review | ✔️ | Cover/Title |
| 2 | Structured abstract | ✔️ | Abstract |
| 3 | Rationale (justification) | ✔️ | Introduction |
| 4 | Clear objectives (PCC) | ✔️ | Introduction / Methods |
| 5 | Protocol registered | ✔️ | Methods (OSF) |
| 6 | Eligibility criteria | ✔️ | Methods (PCC, Inclusion/Exclusion) |
| 7 | Information sources described | ✔️ | Methods (Databases) |
| 8 | Detailed search strategy | ✔️ | Methods / Appendix I |
| 9 | Study selection process | ✔️ | Methods (Rayyan, screening) |
| 10 | Data extraction process | ✔️ | Methods (Zotero, Rayyan) |
| 11 | Data items described | ✔️ | Methods (PCC, parameters assessed) |
| 12 | Description of synthesis methods | ✔️ | Methods (narrative, tables) |
| 13 | Flow diagram of selection process | ✔️ | Results (Figure 1) |
| 14 | Characteristics of included studies | ✔️ | Results (Study design, Formulations) |
| 15 | Results of individual studies | ✔️ | Results (tables, narrative description) |
| 16 | Synthesis of results | ✔️ | Results and Discussion |
| 17 | Discussion of main findings | ✔️ | Discussion |
| 18 | Discussion of study limitations | ✔️ | Discussion (Gaps and implications) |
| 19 | Discussion of review limitations | ✔️ | Section “Methodological Limitations” |
| 20 | Conclusions aligned with objectives | ✔️ | Conclusion |
| 21 | Funding and conflicts of interest declared | ✔️ | Final section (Funding/Conflict) |
| 22 | PRISMA-ScR checklist provided | ✔️ | This document |

**Supplementary Table S2.** Systematic search in the electronic databases PubMed, Scopus, Web of Science, and LILACS realized in June 2025.

| **PubMed/MEDLINE** |
| --- |
| ("*Mentha piperita*"[Title/Abstract] OR "peppermint oil"[Title/Abstract]) AND ("wound"[Title/Abstract] OR "healing"[Title/Abstract] OR "skin"[Title/Abstract] OR "cutaneous"[Title/Abstract] OR "lesion"[Title/Abstract] OR "injury"[Title/Abstract]) AND ("topical"[Title/Abstract] OR "formulation"[Title/Abstract] OR "treatment"[Title/Abstract] OR "cream"[Title/Abstract] OR "gel"[Title/Abstract] OR "nanoemulsion"[Title/Abstract] OR "ointment"[Title/Abstract]) AND ("2010/01/01"[Date - Publication] : "2025/06/05"[Date - Publication]).  Search conducted on June 13, 2025, at 3:28 p.m. – 51 results found |
| **Scopus** |
| (TITLE-ABS-KEY ("*Mentha piperita*" OR "peppermint oil") AND TITLE-ABS-KEY ("wound" OR "healing" OR "skin" OR "cutaneous" OR "lesion" OR "injury") AND TITLE-ABS-KEY ("topical" OR "formulation" OR "treatment" OR "cream" OR "gel" OR "nanoemulsion" OR "ointment").  Search conducted on June 15, 2025, at 4:40 p.m. - 245 results found |
| **LILACS** |
| ("hortelã" OR "*Mentha piperita*" OR "óleo essencial de hortelã" OR "peppermint oil") AND (ferida OR lesão OR pele OR inflamação OR cicatrização OR úlcera OR "wound" OR "injury" OR "skin" OR "healing" OR "inflammation" OR "ulcer").  Search conducted on June 15, 2025, at 3:16 p.m. - 69 results found |
| **WEB OF SCIENCE** |
| ("*Mentha piperita*" OR "peppermint oil") AND ("wound healing" OR "skin" OR "cutaneous" OR "injury" OR "lesion" OR "ulcer" OR "inflammation") AND ("topical application" OR "topical treatment" OR "cream" OR "gel" OR "ointment" OR "nanoemulsion" OR "formulation") |
| Search conducted on June 15, 2025, at 4:15 p.m. - 47 results found |
